# Case of a Compound Fracture of the Leg; with Remarks

**Published:** 1792

**Authors:** Henry Yates Carter

**Affiliations:** Surgeon at Kettley, near Wellington, in Shropshire.


### MEDICAL FACTS

AND

### OBSERVATIONS.

I. *Case of a compound Fracture of the Leg; with Remarks. By Mr.* Henry Yates Carter, *Surgeon at Kettley, near Wellington, in Shropshire.*

ON the 4th of January, 1785, Thomas Oliver, a Collier, sixty years old, received, from the fall of a bucket down a coal pit, in which he was at work, a compound fracture of the bones of his right leg.

The injury was effected with so much violence as to occasion an almost complete division of the muscles; so that the lower part of the leg, with the foot, remained hanging only by a small portion of the gastrocnemius internus or soleus muscle.

When I firſt ſaw him, which was late at night, about two hours after the accident, I found, beſides the laceration of the integuments, muſcles, and other ſoft parts, a conſiderable protruſion of the tibia, in conſequence of the violence of the muſcular contraction; his ſtrength, at the ſame time, ſeeming to be greatly reduced by extreme pain and loſs of blood: ſo that when to theſe appearances I added the circumſtances of his age, and habit of body, (for he had been afflicted with ſcorbutic ulcers in the fractured limb for ſeveral years) I muſt confeſs I had little or no expectation of ſucceſs from any other remedy than the knife. As the hæmorrhage, however, which at firſt had been profuſe, was now conſiderably checked, (probably by his expoſure to cold, for he had been brought a conſiderable diſtance on horſeback) and was ſoon totally ſtopped by the application of ſpirit of wine to the wound, aſſiſted by ſlight preſſure, by means of a tourniquet, on the crural artery, I reſolved to defer the farther conſideration of the caſe till the next morning.

After the wound had been ſufficiently cleanſed, a ſlight extenſion was found ſufficient to reduce the limb to its natural poſture, which was immediately immediately done. The wound was then dref-fed with lint faturated with vinegar and tincture of myrrh, and the limb being fecured in its fituation, and an anodyne adminiftered to the patient, I left him, under proper care, for the night; having determined in my own mind to propofe, the next morning, either the amputation of the leg, or his removal, if poffible, to the County Infirmary, as the poor man's fituation, together with his having a large and helplefs family, appeared to be forcible reafons for his removal, and to operate againft the probability of his recovery at home.

The next day (January the 5th) I vifited him early, and found that he had refted much better than could reafonably have been expected. He had but little fever, and appeared to be in tolerable fpirits. The difcharge of blood, from the time I had left him, had been fo inconfiderable as not to have come through the dreffings; and as the limb did not feem to fuffer from the extremely gentle ftricture of the tourniquet, it was thought advifable to leave it on the limb.

As the patient ftrenuoufly objected to the propofal of removing him to the Infirmary, as well as to that of amputation, it became neceffary to attempt the cure of the limb at home without an operation.

The firft thing to be confidered was how to fecure a limb in fuch a ftate in perfect contact; and in the next place how to guard againft an accumulation of matter between the divided ends of the bone: for it was effential that the limb fhould be kept perfectly fteady, and it was reafonable to fuppofe that fo large a wound would, from the difcharge, require frequent dreffing. I firft began, therefore, by preparing a flexible wooden fplint, covered with leather, about four inches broad and ten long, upon which was laid a very thick comprefs or bolfter of linen of the fame dimenfions, the lower part of which was filled with tow, in order to remedy the inequality of the fmall part of the leg: upon this was laid an eighteen-headed bandage, the two external leaves of which were ftiffened with white of eggs, in order, in fome meafure, to ferve as a fubftitute for fplints, in the ordinary mode of application, which in this cafe was inadmiffible. Thefe arrangements being made, the limb was carefully raifed while an affiftant placed the fplint, &c., under it on a pillow, upon which the limb was laid without any inconvenience or pain. None of

3 the the dreſſings before employed were removed, except the external layer of lint, a freſh quantity of which was applied, and the bandage laid regularly over it as tight as the nature of the caſe would admit, an opening through the center leaves being made for the admiſſion of all applications that might be neceſſary, as I was determined to leave the dreſſings undiſturbed as long as poſſible. A looſe ſplint was then applied, without any ligature, on each ſide of the limb, and ſecured in that poſture by bolſters of cloth, which were continued on each ſide above the knee.

A liquid application was then ordered to be very frequently applied, compoſed of camphorated ſpirit of wine, two ounces; vinegar, four ounces; tincture of myrrh, one ounce, and ſal ammoniac, a ſcruple: the direction for the uſe of which was ſtrictly adhered to by the perſon who was employed to be conſtantly with the patient, in order to prevent any accident happening to the limb during ſleep.

A powder, containing thirty grains of vitriolated tartar, and twenty of diaphoretic antimony, was directed to be adminiſtered to him every third hour, in barley water, or ſome other ſuitable vehicle; and at night a draught was given containing twenty-five drops of laudanum.

On the 6th, finding the ſymptoms of fever rather increaſed, I gave him a purgative draught, which procured two ſtools. He had reſted tolerably well; the limb continued ſecure: he began to complain of a ſenſe of ſoreneſs and of ſhooting (to uſe his own expreſſion) in the limb. In other reſpects he was as well as could be expected, The uſe of the lotion was continued without any alteration.

There was little or no material change in the general circumſtances of the caſe till the 10th, when, upon finding ſome increaſing ſymptoms of fever, which I had reaſon to think were kept up, if not occaſioned, by coſtiveneſs, I repeated the purgative medicine, and with a good effect.

On the 11th, obſerving appearances of diſcharge from the dreſſings, they were carefully taken off; the pus ſeemed to be favourable, and the wound was in a granulating ſtate. A freſh bandage was, without much difficulty, drawn under the limb; and the dreſſings and other applications, with little variation, were continued as before till the 17th, when the wound was again opened, and I had the ſatisfaction to find a callus forming, and the wound in good condition.

As the discharge from the wound was now considerable, and the patient complained of thirst and that his appetite was not so good as it had been, I advised a liberal use of Port wine, and of the red Peruvian bark, both in substance and in decoction, to which I added small doses of nitre, till the 20th, when the symptoms of fever seemed to have subsided. The wound was again dressed on that day, and from that time regularly every day.

After the 30th, the use of the bark seeming to be no longer indicated, it was wholly omitted; the bones gradually united, and the wound healed without any interruption, except from a few small splinters of bone, which came away without much difficulty, and did not appear to be exfoliations.

By the 10th of March he was able to leave his bed, and to walk with the assistance of crutches, and from that time I ceased to attend him. The wound was then completely healed, forming a firm cicatrix; and the leg was straight.

This extraordinary fact is a more ftriking proof than any I have ever met with of the neceffity there is for great deliberation in cafes where amputation may be thought neceffary on account of recent external injuries. As a caution to us in cafes of this kind, and to fhow that we ought not precipitately to have recourfe to extirpation even in the worft kind of fractures, Van Swieten* has quoted from De la Motte a cafe, fomewhat fimilar to mine, of a man who had the tibia and fibula fractured by the wheels of a carriage loaded with feveral thoufand weight paffing over his leg, which occafioned fuch a contufion and laceration of the part, that the whole limb might have been eafily divided with one cut or two of the fciffars. In that cafe a large portion of the tibia was feparated, but, notwithftanding this circumftance, the patient, we are told, gradually recovered the ufe of his limb, without any deformity remaining.

I am aware that Mr. Pott, whofe opinion is defervedly of high authority in furgery, particularly refers to the cafe juft now quoted from De la Motte, and obferves, that in his opinion,

* Comment. in Aph. Boerh. § 351.

the furgeon fhewed much more rafhnefs in attempting to fave fuch a limb, than he would have done in the amputation of it*. In the cafe of my patient, however, there could be no ground for fuch a cenfure; for I am ready to confefs that nothing but his abfolute refufal to fubmit to the operation prevented me from taking off the limb. How far Mr. Pott's arguments† in favour of fpeedy amputation, in cafes of this fort, are well founded, I fhall not at prefent attempt to difcufs; but however juftifiable an operation might have been under circumftances fo alarming as thofe I have defcribed, and in which there appeared fuch little probability of fuccefs, I cannot help thinking that, in fimilar cafes, where hæmorrhage can by any fafe means be checked, the divided parts brought into and retained in contact, and when the vital powers have not been too much exhaufted, we fhould be deterred neither by the age of the patient, nor by a feemingly bad habit of body, (reafons that, to my knowledge, are too

* Remarks on Fractures and Diflocations. 8vo. London, 1769. p. 75.

† Ibid. p. 73 & feq.

often often alledged) from endeavouring to avoid the neceſſity of having recourſe to amputation.

Upon the whole, I feel myſelf diſpoſed to join in opinion with Dr. Kirkland*, that, however neceſſary and right ſpeedy amputation may be in great hoſpitals, this ought to be no precedent for country practice, in which much more may certainly be expected from the reſources of nature than many imagine.—" My " connections," obſerves the experienced and judicious writer juſt now mentioned, " with " thoſe of my profeſſion have not only led " me to know the ſucceſs of many Surgeons, " whoſe ſituation affords them only common " accidents; but alſo of ſeveral, who,as well as " myſelf, have had the care of the workmen in " Collieries, Lime-kilns, Lead-mines, and the " like, where the moſt violent injuries of this " kind frequently happen. In theſe places, the " bones are, for the moſt part, not only broken " into many pieces, and their extremities, now " and then, ſeparated, ſo as to come away; " but they are alſo often forced into the ground, " the principal arteries ſometimes divided, and

* Obſervations upon Mr. Pott's General Remarks on Fractures, &c. 8vo. London, 1770. p. 41.

" the

“ the muſcles, &c. are frequently lacerated, and

“ cruſhed with immenſe weights, even ſo much,

“ that coal ſleck, &c. in great quantities, is

“ driven into the very ſubſtance of the fleſh,

“ ſo as to render the accident as formidable as

“ poſſible; and yet, it is a notorious fact, that,

“ where the part is not abſolutely deſtroyed,

“ theſe deſperate caſes ſeldom fail of being

“ cured, without the loſs of the limb *.”

*Kettley,*

Auguſt 2, 1791.

